# Comprehensive insights into AML relapse: genetic mutations, clonal evolution, and clinical outcomes

**DOI:** 10.1186/s12935-024-03368-4

**Published:** 2024-05-19

**Authors:** Namsoo Kim, Seungmin Hahn, Yu Jeong Choi, Hyunsoo Cho, Haerim Chung, Ji Eun Jang, Chuhl Joo Lyu, Seung-Tae Lee, Jong Rak Choi, June-Won Cheong, Saeam Shin

**Affiliations:** 1 Department of Laboratory Medicine, Yonsei University College of Medicine, Severance Hospital, 50 Yonsei-ro, Seodaemun-gu, Seoul, 03722 Korea; 2 Department of Pediatric Hematology-Oncology, Severance Hospital, Yonsei Cancer Center, Yonsei University College of Medicine, Seoul, Korea; 3 Division of Hematology, Department of Internal Medicine, Yonsei University College of Medicine, Severance Hospital, 50 Yonsei-ro, Seodaemun-gu, Seoul, 03722 Korea; 4 Dxome Co. Ltd, Seongnam-si, Gyeonggi-do Korea

**Keywords:** Acute myeloid leukemia, Relapse, Next-generation sequencing, Gene rearrangement, RNA sequencing

## Abstract

**Introduction:**

Acute myeloid leukemia (AML) is a complex hematologic malignancy characterized by uncontrolled proliferation of myeloid precursor cells within bone marrow. Despite advances in understanding of its molecular underpinnings, AML remains a therapeutic challenge due to its high relapse rate and clonal evolution.

**Methods:**

In this retrospective study, we analyzed data from 24 AML patients diagnosed at a single institution between January 2017 and August 2023. Comprehensive genetic analyses, including chromosomal karyotyping, next-generation sequencing, and gene fusion assays, were performed on bone marrow samples obtained at initial diagnosis and relapse. Clinical data, treatment regimens, and patient outcomes were also documented.

**Results:**

Mutations in core genes of *FLT3*, *NPM1*, *DNMT3A*, and *IDH2* were frequently discovered in diagnostic sample and remained in relapse sample. *FLT3*-ITD, *TP53*, *KIT*, *RUNX1*, and *WT1* mutation were acquired at relapse in one patient each. Gene fusion assays revealed stable patterns, while chromosomal karyotype analyses indicated a greater diversity of mutations in relapsed patients. Clonal evolution patterns varied, with some cases showing linear or branching evolution and others exhibiting no substantial change in core mutations between diagnosis and relapse.

**Conclusions:**

Our study integrates karyotype, gene rearrangements, and gene mutation results to provide a further understanding of AML heterogeneity and evolution. We demonstrate the clinical relevance of specific mutations and clonal evolution patterns, emphasizing the need for personalized therapies and measurable residual disease monitoring in AML management. By bridging the gap between genetics and clinical outcome, we move closer to tailored AML therapies and improved patient prognoses.

**Supplementary Information:**

The online version contains supplementary material available at 10.1186/s12935-024-03368-4.

## Introduction

Acute myeloid leukemia (AML) is a heterogeneous and aggressive hematologic malignancy characterized by uncontrolled proliferation of myeloid precursor cells within the bone marrow [1]. Despite advancements in the understanding of its molecular pathogenesis, AML remains challenging to manage, with a high propensity for relapse following initial therapy [2]. Relapsed AML often exhibits genetic and clonal evolution, further complicating its diagnosis and treatment [3]. Like other cancers, relapsed AML has a poor prognosis, and treatment options are also challenging.

To identify genetic mutations in AML, various methods have been developed. The historic approach is chromosomal karyotyping [4], through which it is possible to determine if there are recurrent mutations and to identify chromosome gains or losses [5]. From chromosomal karyotyping, typical abnormalities such as deletion 5, deletion 7, inversion 16, and t(15;17) can be detected. To examine mutations at the gene level, next-generation sequencing (NGS) has emerged as a prominent method [6]. Clinical laboratories use panels targeting specific genes for testing [7], while research studies may involve whole exome sequencing or whole genome sequencing [8]. Through methods such as NGS, mutations in genes such as *FLT3*, *NPM1*, and *DNMT3A* can be detected. Since it determines whether there is a mutation in specific codons, it is advantageous for patient-specific minimal residual disease follow-up. NGS allows the identification of gene amplifications, deletions, as well as chromosomal gains and losses but may fall short in identifying gene breakpoints [9]. Therefore, RNA-based gene fusion assays have been developed [10]. In most clinical laboratories, tests are conducted for genes with common known breakpoints in acute leukemia, and these results can be used for precise diagnosis [11]. RNA-based sequencing is also being actively researched recently, and it is suitable for detecting various types of gene fusions in recent AML diagnostic guidelines. Recently, optical mapping methods have been employed to perform comprehensive testing for all genes [12].

Historically, the prognosis for acute myeloid leukemia has been quite poor, and there have been extensive discussions regarding relapse and post-relapse treatment [13]. Research using NGS methods has focused on identifying driver mutations and understanding how clonal evolution progresses based on these drivers [14]. Recent studies have suggested the existence of linear evolution and branching evolution when specific AML clones survive during remission to later contribute to relapse [15]. Linear evolution refers to the gradual accumulation of individual mutations, while branching evolution is characterized by the elimination of the predominant clone, succeeded by the emergence and expansion of a subclone.

Furthermore, while several studies have reported NGS results based on samples collected at AML diagnosis and relapse, our comprehensive analysis revealed certain limitations in their findings. First, they did not present RNA gene fusion results, which can yield positivity rates as high as 60% [16]. Second, there was a lack of chromosome results considered significant in the diagnostic criteria for AML. Third, these studies did not provide information regarding the treatment regimens employed for AML patients and the associated outcomes. In that regard, we believe it is necessary to integrate and analyze multiple tests for relapsed AML patients.

In this study, we aimed to broaden our knowledge of relapsed AML by examining patients who underwent NGS diagnosis and treatment at a single institution, encompassing treatment progress, prognosis, and various other tests. We sought to understand how clonal evolution occurs in these patients as well as which mutations persist through relapse to provide a more comprehensive understanding of AML.

## Methods

### Patients and data

This retrospective study focused on patients diagnosed with AML at a single institution from January 1, 2017, to August 31, 2023, who underwent NGS analysis on bone marrow samples at the time of initial diagnosis and again when experiencing relapse. Clinical data, laboratory test results, and genetic analysis results were collected and analyzed. The data included bone marrow aspiration results and flow cytometry findings at the time of AML diagnosis, as well as details of the treatments received. The initial diagnosis was determined according to WHO guidelines [17], considering flow cytometry, chromosome karyotyping, gene fusion, and NGS results. In cases of AML relapse, information was collected regarding the duration to relapse, clinical data and laboratory test results at the time of relapse, treatments received, and current prognosis. Patient outcomes were categorized as alive, dead, or follow-up loss. This study protocol received approval from the Institutional Review Board of Yonsei University Health System (4-2023-0930).

### Cytogenetic and molecular genetic analyses

The G-banding karyotyping procedure followed standard protocols and was performed on heparinized bone marrow aspirate. A minimum of 20 metaphases was assessed, and the karyotype was described in accordance with the International System for Human Cytogenetic Nomenclature.

During the initial diagnosis, bone marrow aspirate samples were collected in EDTA vials. The QIAamp RNA Blood Mini Kit (Qiagen, Hilden, Germany) was used to extract total RNA for NGS testing. The Agilent 4200 TapeStation System (Agilent Technologies Inc, Santa Clara, CA) was used to measure the quantity and quality of RNA. The Archer FusionPlex Pan-Heme kit (ArcherDX, Boulder, CO) manufacturer’s instructions were followed to prepare a target-enriched cDNA library. Starting with 100 ng of RNA, random primers were used to provide random start sites for the initial cDNA synthesis. The end-repaired cDNA molecules were then ligated with sample-specific indices and specific molecular barcode adapters at both ends. A total of 199 target genes was covered by gene-specific primers used in two rounds of low-cycle PCR. On the NextSeq 550Dx instrument (Illumina, San Diego, CA), the finished products underwent sequencing, producing roughly three million reads per sample over 151 cycles. Next, default parameters were used with Archer Analysis Software (version 5.1, ArcherDX) to process and analyze the raw data, including extraction and evaluation of gene fusions, oncogenic variations, oncogenic isoforms, and mRNA expression data.

For targeted NGS, a customized set of probes (Dxome Co. Ltd., Gyeonggi-do, Korea) targeting 213, 497, or 531 genes associated with hematologic neoplasms was utilized (Supplementary Table S1). Genomic DNA extracted from the diagnostic bone marrow aspirate was used for library construction. These libraries underwent hybridization with capture probes and were sequenced using Illumina’s NextSeq 550Dx (San Diego, CA, USA), following the manufacturer’s guidelines. NGS data analysis was carried out using the DxSeq analyzer (Dxome). Single-nucleotide variants, small insertions and deletions, and copy number variants were detected using established methods [18, 19]. Variant allele frequency (VAF) represents the proportion of sequence reads that align with a particular DNA variant, divided by the total coverage observed at that specific genomic location. Validation of germline variants was conducted by comparing NGS results with buccal swabbing or skin fibroblast analysis. The identified variants were classified into four tiers based on the Association for Molecular Pathology’s guidelines, which incorporate input from the American College of Medical Genetics, the American Society of Clinical Oncology, and the College of American Pathologists [20]. During classification, web databases such as OncoKB [21] and cBioPortal [22] were consulted. The accuracy of all variants was visually confirmed using the Integrated Genomics Viewer.

### Data availability

All data generated or analyzed during this study are included in this published article and its supplementary information files.

## Results

### Patient demographics

A total of 24 patients, 10 male and 14 female, was enrolled in this study (Table 1). The ages of the patients ranged from 2 to 72 years. The initial diagnosis was determined by integrating the results of flow cytometry and bone marrow aspiration, resulting in 8 different AML diagnoses: acute megakaryoblastic leukemia, acute monocytic leukemia, acute myeloid leukemia with maturation, acute myeloid leukemia with mutated *NPM1*, acute myeloid leukemia with t(16;16)(p13.1;q22), acute monocytic leukemia, acute myeloid leukemia with t(8;21)(q22;q22.1), and acute myelomonocytic leukemia. Among the patients, 15 received hematopoietic stem cell transplantation (HSCT) during their initial treatment, while 9 underwent only chemotherapy. The average time to relapse was 14 months (ranging from 5 months to 2 years and 7 months). Among the relapsed patients, 11 received HSCT, 10 underwent chemotherapy only, and 3 did not receive any treatment. Among the HSCT-treated patients, 7 of 11 survived, while 4 succumbed to the disease. Among the patients who underwent chemotherapy alone, 8 died, and 2 were lost to follow-up. Among the 3 patients who did not receive treatment, 2 died, and 1 was lost to follow-up.

**Table 1:** Patient characteristics

P#	Sex	Age of diagnosis	Initial Diagnosis	Treatment at diagnosis	Time to relapse	Treatment at relapse	Prognosis
P1	M	2Y11M	Acute megakaryoblastic leukemia	CTx	1Y9M	CTx	Follow-up loss/1Y7M
P2	F	2Y9M	Acute monocytic leukemia	CTx, HSCT	1Y	CTx	Dead/5 M
P3	M	63Y	Acute myeloid leukemia with maturation	CTx, HSCT	2Y2M	CTx, HSCT	Dead/10 M
P4	F	60Y	Acute myeloid leukemia with mutated *NPM1*	CTx, HSCT	1Y	CTx	Dead/7 M
P5	F	54Y	Acute myeloid leukemia with mutated *NPM1*	CTx, HSCT	7 M	CTx	Dead/3 M
P6	M	51Y	Acute myeloid leukemia with t(16;16)(p13.1;q22)	CTx	11 M	CTx, HSCT	Alive/3Y5M
P7	F	59Y	Acute monocytic leukemia	CTx, HSCT	5 M	CTx	Dead/1Y2M
P8	F	55Y	Acute myeloid leukemia with t(8;21)(q22;q22.1)	CTx, HSCT	1Y6M	CTx	Follow-up loss/4 M
P9	F	61Y	Acute myeloid leukemia with mutated *NPM1*	CTx	8 M	CTx	Dead/6 M
P10	M	65Y	Acute myeloid leukemia with t(8;21)(q22;q22.1)	CTx	1Y	CTx, HSCT	Dead/1Y1M
P11	F	58Y	Acute myeloid leukemia with biallelic mutations of *CEBPA*	CTx	2Y6M	CTx, HSCT	Alive/11 M
P12	F	20Y	Acute myeloid leukemia with maturation	CTx, HSCT	1Y3M	CTx, HSCT	Alive/2Y2M
P13	F	44Y	Acute myeloid leukemia with maturation	CTx	2Y7M	CTx, HSCT	Alive/10 M
P14	M	72Y	Acute myeloid leukemia with mutated *NPM1*	CTx	1Y1M	None	Follow-up loss/1 M
P15	F	58Y	Acute myelomonocytic leukemia	CTx, HSCT	1Y5M	CTx	Dead/2 M
P16	M	62Y	Acute myeloid leukemia with t(8;21)(q22;q22.1)	CTx, HSCT	7 M	None	Dead/1 M
P17	M	63Y	Acute myelomonocytic leukemia	CTx, HSCT	7 M	CTx	Dead/2 M
P18	M	40Y	Acute monocytic leukemia	CTx, HSCT	6 M	CTx, HSCT	Alive/2Y
P19	F	49Y	Acute myeloid leukemia with t(8;21)(q22;q22.1)	CTx	2Y1M	CTx, HSCT	Alive/3 M
P20	M	35Y	Acute myeloid leukemia with mutated *NPM1*	CTx, HSCT	8 M	CTx, HSCT	Dead/6 M
P21	F	2Y2M	Acute myeloid leukemia with maturation	CTx, HSCT	1Y6M	CTx, HSCT	Alive/7 M
P22	F	73Y	Acute myeloid leukemia with biallelic mutations of *CEBPA*	CTx	10 M	CTx	Dead/5 M
P23	M	61Y	Acute monocytic leukemia	CTx, HSCT	10 M	CTx, HSCT	Dead/9 M
P24	F	62Y	Acute myeloid leukemia with maturation	CTx, HSCT	1Y2M	None	Dead/1 M

Abbreviations: P: patient; CTx: chemotherapy; HSCT: hematopoietic stem cell transplantation; Y: year; M: month

### Oncogenic mutations

**Fig. 1:**
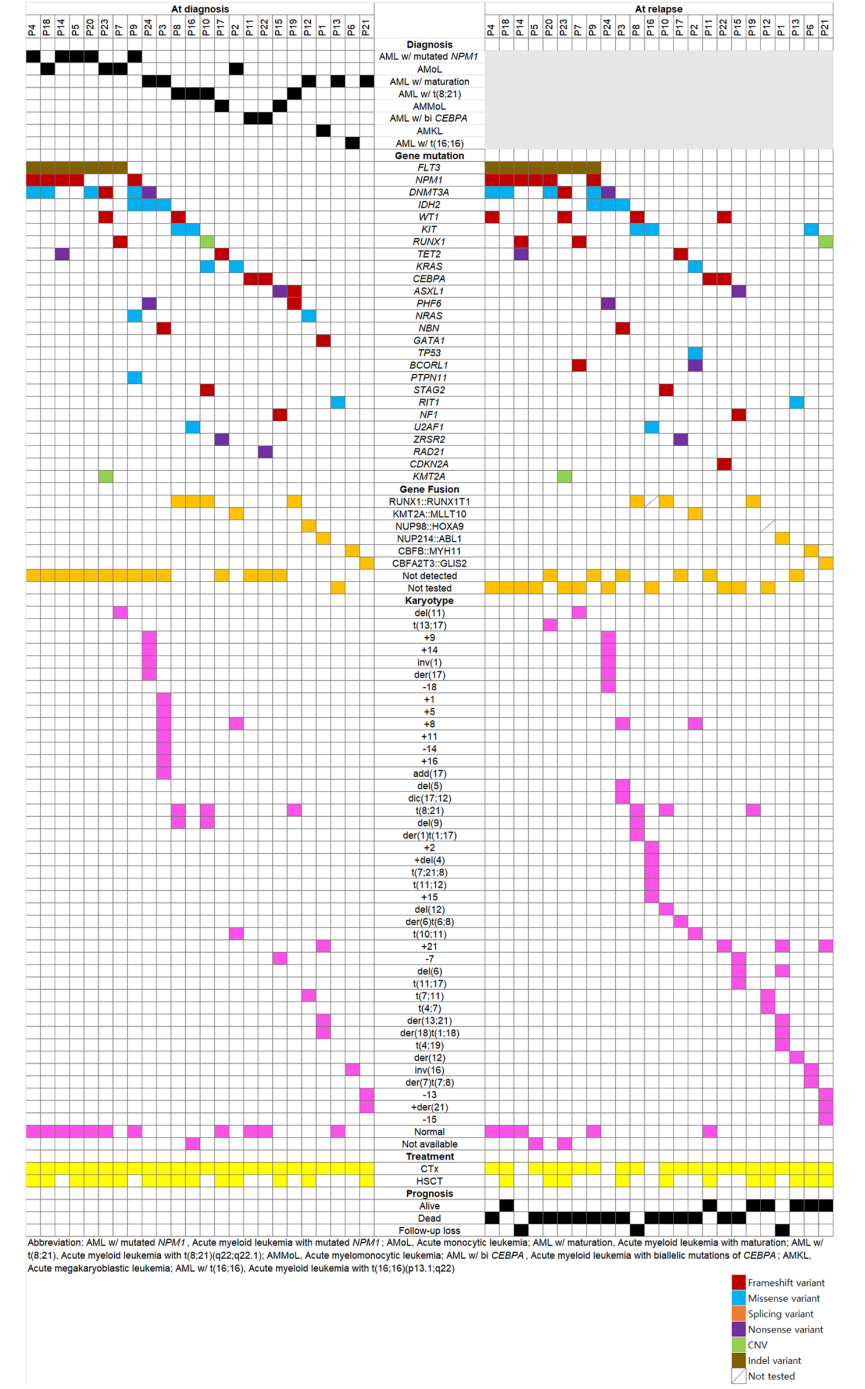
Oncoplot of the case distribution of relapsed acute myeloid leukemia patients

To identify oncogenic mutations in the patients, three main tests were conducted for cytogenetics and molecular genetics: NGS, gene fusion analysis, and chromosome karyotyping. The average depth for NGS was 1346.3x (from 407.4x to 3648.0x). The results of these tests were correlated with treatment and prognosis (Fig. 1). Among the core gene mutations identified by NGS, the most frequent genes were *FLT3*, *NPM1*, *DNMT3A*, and *IDH2*, in that order. In the case of *FLT3* mutations, 7 patients had mutations at diagnosis, and all 7 maintained the mutations at relapse; the 8th patient presented with the mutation at relapse. The core gene mutations identified as tier 1/2 occurred on 26 genes, present in panels ranging from the smallest 213 gene panel to the largest 531 gene panel.

Twelve patients underwent a fusion test both at diagnosis and relapse and seven patients were fusion positive. Gene fusions detected included *RUNX::RUNX1T1, KMT2A::MLLT10, NUP98::HOXA9, NUP214::ABL1, CBFB::MYH11*, and *CBFA2T3::GLIS2* fusions. Among them, *RUNX1::RUNX1T1* fusion was detected in 4 patients, while the other gene fusions were detected in 1 patient each. All gene fusions remained stable upon relapse detection, with no new fusions gained or lost. Chromosome karyotype analysis revealed various types of abnormalities, with relapsed patients often exhibiting a greater diversity of abnormalities compared to their diagnostic samples.

**Fig. 2:**
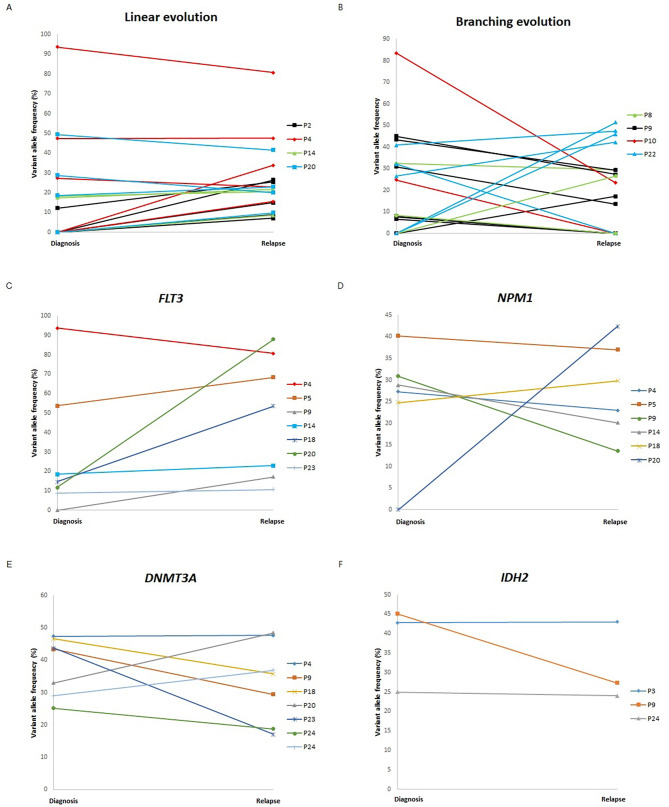
The changes of variant allele frequencies in (**A**) linear evolution patients’ tier 1,2 and 3 variants and (**B**) branching evolution patients’ tier 1,2 and 3 variants for (**C**) *FLT3*, (**D**) *NPM1*, (**E**) *DNMT3A*, and (**F**) *IDH2*.

Analyses of 24 patients regarding the correspondence of tier 1/2 core mutations to evolutionary models are described in Table 2. Five patients experienced linear evolution, 4 with branching evolution, and 15 were classified as unclear. Among the 15 unclear cases, 11 exhibited an exact match in core mutations between diagnosis and relapse. Among the 24 patients and the 26 core mutation genes, 9 were categorized as linear or branching evolution. The 4 most frequent core genes (*FLT3, NPM1, DNMT3A, IDH2*) were compared in terms of VAF between diagnosis and prognosis (Fig. 2). Additionally, graphs were generated for the variation in VAF across tiers 1, 2, and 3 for patients classified under linear evolution and branching evolution. Linear evolution was characterized by a high occurrence of new mutations, while the VAF of pre-existing mutations remained relatively consistent or slightly decreased. In contrast, branching evolution often featured decreased VAF for pre-existing mutations, with fewer new mutations compared to linear evolution. All 4 core genes that were present at diagnosis were also present at relapse, except for 1 patient who acquired an *FLT3* mutation at relapse and 1 who acquired an *NPM1* mutation at relapse. *FLT3* mutations exhibited increased VAF at relapse in all patients except 1, while a consensus on VAF changes for *NPM1*, *DNMT3A*, and *IDH2* was not observed. All variants identified by NGS panels are provided in Supplementary Table S2.

**Table 2:** Core gene mutations in AML patients

*P*#	Diagnosis	Relapse	Model
P1	*GATA1*	ND	Unclear
P2	*KRAS*	*TP53, KRAS, BCORL1*	Linear evolution
P3	*IDH2, NBN*	*IDH2, NBN*	Unclear
P4	*DNMT3A, NPM1, FLT3*	*DNMT3A, NPM1, FLT3, WT1*	Linear evolution
P5	*FLT3, NPM1*	*FLT3, NPM1*	Unclear
P6	ND	*KIT*	Unclear
P7	*FLT3, RUNX1*	*FLT3, BCORL1, RUNX1*	Linear evolution
P8	*KIT, WT1*	*KIT, WT1*	Branching evolution
P9	*NPM1, IDH2, DNMT3A, PTPN11, NRAS*	*FLT3, NPM1, IDH2, DNMT3A*	Branching evolution
P10	*RUNX1, KRAS, STAG2*	*STAG2*	Branching evolution
P11	*CEBPA*	*CEBPA*	Unclear
P12	*NRAS*	ND	Unclear
P13	*RIT1*	*RIT1*	Unclear
P14	*FLT3, NPM1, TET2*	*FLT3, NPM1, TET2, RUNX1*	Linear evolution
P15	*ASXL1, NF1*	*ASXL1, NF1*	Unclear
P16	*KIT, U2AF1*	*KIT, U2AF1*	Unclear
P17	*TET2, ZRSR2*	*TET2, ZRSR2*	Unclear
P18	*FLT3, NPM1, DNMT3A*	*FLT3, NPM1, DNMT3A*	Unclear
P19	*ASXL1, PHF6*	ND	Unclear
P20	*FLT3, DNMT3A*	*FLT3, DNMT3A, NPM1*	Linear evolution
P21	ND	*RUNX1*	Unclear
P22	*CEBPA, RAD21*	*CEBPA, WT1, CDKN2A*	Branching evolution
P23	*FLT3, KMT2A, WT1, DNMT3A*	*FLT3, KMT2A, WT1, DNMT3A*	Unclear
P24	*DNMT3A, IDH2, PHF6*	*DNMT3A, IDH2, PHF6*	Unclear

Abbreviations: P: patient; ND: not detected

## Discussion

In this study, we present comprehensive results of a cohort of 24 patients diagnosed with AML. Through this study, we were able to integrate chromosomal karyotyping, gene fusion results, and NGS findings. The findings from this study contribute to our understanding of AML heterogeneity and evolution, providing valuable insights for future therapeutic strategies.

Oncogenic mutations play a pivotal role in AML pathogenesis and progression [23]. Our investigation into the mutational landscape through cytogenetic and molecular genetic analyses revealed several significant insights. The most frequently observed core mutations, *FLT3*, *NPM1*, *DNMT3A*, and *IDH2*, mostly remained consistent between diagnosis and relapse stages. Mutations in *DNMT3A*, *ASXL1*, and *RUNX1* genes and *FLT3*-ITD were known to be frequently acquired at relapse [15]. In our study, *FLT3*-ITD, *TP53*, *KIT*, *RUNX1*, and *WT1* mutation were acquired at relapse in one patient each. These genes are associated with a poor prognosis of AML [24–27] and mutations in these genes have been reported to be AML relapse-associated mutations in previous studies [28–30]. Therefore, they are presumed to be associated with the progression and treatment resistance of AML.

In our study, fusion results showed that mutations present at diagnosis remained unchanged at relapse. These findings suggest that the gene rearrangements were early events in these cases. However, performing a gene fusion test at diagnosis and relapse might be helpful when referring to existing reports that *BCR::ABL1* can be required in AML relapse [31]. Unlike gene fusions, chromosomal karyotyping revealed various alterations, including gains, losses, and translocations, acquired by the chromosomes at relapse. Our finding supports that chromosomal instability acts as a mechanism for AML relapse, as previously suggested [32].

In our research, the patient cohort was highly diverse and was classified into four groups: pediatric (under 15 years old), adolescents and young adults (from 15 to 29 years old), adults (30 to 64 years old), and elderly (65 years and older), which consisted of 3, 1, 18, and 2 patients, respectively. The adult group had the largest number of patients, and survivors were distributed only among the adolescents and young adults and adult groups. Notably, four patients belonging to the pediatric or adolescents and young adults groups showed a characteristic feature of gene rearrangement detection in targeted RNA sequencing. This observation is in agreement with recent diagnostic trends, where the presence of specific gene rearrangements can lead to a change in the diagnosis of AML and plays a significant role in prognosis [27]. Branching evolution was found to occur in the higher age bracket of those aged 55 to 73 years. indicating that branching evolution occurs more frequently in the elderly population. These patients exhibited a pattern of certain remaining clones after anticancer treatment. If relapse is predicted after chemotherapy due to the persistence of specific clones, alternative chemotherapy regimens may be necessary.

The importance of MRD is being emphasized, and an ideal MRD technique in AML must be established [33]. In our study, four patients were followed up with reverse-transcription PCR (RT-PCR). RT-PCR detected morphological relapse at three months (P6) and four months (P10) before morphological relapse or continued to be RT-PCR positive during morphological complete remission (P8 and P19), suggesting the usefulness of MRD monitoring using RT-PCR. However, gene fusion was not detected in more than half of our patients. Therefore, NGS-based MRD might be helpful in these patients. In addition, considering that 50% of the total 24 patients showed clonal evolution with emerging or vanished mutations, our results suggest that the agnostic panel approaches might be more valuable than the patient-specific panel among NGS-based MRD strategies in these patients.

This study has several limitations. First, it is a retrospective study conducted in a single institution, so sample size is small, and there might be potential biases in patient selection. However, our findings highlight the complex interplay of genetic and clonal factors in AML, emphasizing the need for larger studies to validate these observations. Second, our study could only identify some of the molecular patterns that appear in the diagnosis and relapse of AML because we used targeted panel sequencing. Previous studies have used whole exome or whole genome sequencing to determine molecular profiles associated with AML relapse [3, 28]. Moreover, recent studies used single-cell analysis to reveal the pathogenic mechanisms of AML relapse [34, 35]. With the advancement of genomic techniques, more information has been available to determine the precise mechanisms of chemoresistance and relapse of AML. However, our study suggests that the gene panel sequencing currently available in routine diagnostics without using complex genomic techniques may help identify changes in genomic profiles associated with the recurrence of AML in a clinical setting. Third, we included only samples of diagnosis and relapse in this study. Studies including complete remission samples need to determine how much earlier the AML relapse can be predicted through molecular studies [36]. Whether the mutations found at diagnosis were cleared at complete remission is also important in predicting the patient’s prognosis [37].

## Conclusions

We tried to identify the molecular feature associated with the progression of AML by comparing chromosome abnormalities, gene rearrangements, and gene mutation results at diagnosis and relapse. Patients showed branching and linear evolution patterns; some patients were not clearly classified. Acquired mutations at relapse are likely to play a role in the chemoresistance of AML. Moreover, we could suggest evidence on the optimal MRD monitoring strategy of AML through our study result. Since mutations at the time of diagnosis are not maintained at relapse and disappear or newly emerge in about half of patients, gene panel-based MRD monitoring would be helpful for AML patients.

While our findings contribute to understanding AML heterogeneity, acknowledging limitations like the small sample size, future research should focus on refining MRD measurement methods and exploring comprehensive diagnostic approaches for more effective therapeutic strategies. Overall, our study lays the groundwork for further investigations to enhance the management of relapsed AML patients.

### Electronic supplementary material

Below is the link to the electronic supplementary material.

Supplementary Material 1

## Data Availability

All data generated or analysed during this study are included in this published article and its supplementary information files.
