# Supplementary material for: Comprehensive insights into AML relapse: genetic mutations, clonal evolution, and clinical outcomes
Source: Cancer Cell Int. 2024 May 19;24:174. doi: 10.1186/s12935-024-03368-4 (PMC11103850; doi:10.1186/s12935-024-03368-4)
Supplement: Supplementary file 1 — Supplementary Material 1 [file 12935_2024_3368_MOESM1_ESM.docx]

**Supplementary Table S1.** List of the genes utilized in the study's gene panel.

1. 213 genes

*ABCB7, ABL1, ACD, ADA, AK2, ANKRD26, AP3B1, ASXL1, ATM, ATR, ATRX, BCOR, BCORL1, BLM, BRAF, BRCA1, BRCA2, BRCC3, BRINP3, BRIP1, C17orf97, CALR, CBL, CBLB, CBLC, CDAN1, CDKN2A, CEBPA, CREBBP, CSF2RA, CSF3R, CTC1, CTCF, CUX1, CXCR4, DAXX, DCLRE1C, DDX41, DKC1, DNMT1, DNMT3A, EED, EGFR, EGLN1, ELANE, EPAS1, EPOR, ERCC4, ETNK1, ETV6, EZH2, FAM47A, FANCA, FANCB, FANCC, FANCD2, FANCE, FANCF, FANCG, FANCI, FANCL, FANCM, FAT1, FBXW7, FLRT2, FLT3, G6PC3, GATA1, GATA2, GFI1, GJB3, GNAS, GNB1, GPRC5A, HAX1, HNRNPK, HRAS, IDH1, IDH2, IFNG, IKZF1, IL2RG, IRF1, JAGN1, JAK1, JAK2, JAK3, KAT6A, KCNA4, KCNK13, KDM6A, KDR, KIF23, KIT, KLF1, KMT2A, KMT2C, KRAS, LAMB4, LAMTOR2, LIG4, LRRC4, LUC7L2, LYST, MAP2K1, MPL, MRE11A, MYC, MYD88, NBN, NCOR2, NF1, NHP2, NOP10, NOTCH1, NPM1, NRAS, NSD1, NTRK3, OR13H1, OR8B12, P2RY2, PALB2, PARN, PAX5, PCDHB1, PDGFRA, PHF6, PIGA, PML, POT1, PRAMEF2, PRF1, PRPF40B, PTEN, PTPN11, RAB27A, RAC1, RAC2, RAD21, RAD50, RAD51, RAD51C, RB1, RBM8A, RMRP, RNF168, RPL10, RPL11, RPL15, RPL26, RPL35A, RPL5, RPS10, RPS14, RPS15, RPS17, RPS19, RPS24, RPS26, RPS7, RTEL1, RUNX1, SAXO2, SBDS, SBF2, SEC23B, SETBP1, SF1, SF3A1, SF3B1, SH2B3, SLC37A4, SLX4, SMC1A, SMC3, SRP72, SRSF2, STAG1, STAG2, STX11, STXBP2, SUZ12, SYNE1, TAZ, TCIRG1, TERC, TERT, TET1, TET2, TINF2, TP53, TUBA3C, U2AF1, U2AF2, UBE2T, USB1, VHL, VPS13B, VPS45, WAS, WRAP53, WT1, XRCC2, ZRSR2, UNC13D, XIAP, SH2D1A, TSR2, HBB, SOS1, RAF1, RIT1*

1. 497 genes

*ABCB1, ABCB7, ABCG2, ABCG5, ABCG8, ABL1, ABL2, ACD, ACTB, ACTN1, ADA, ADAMTS13, AIRE, AK1, AK2, AKT2, ALAS2, ALDOA, AMN, ANK1, ANKRD26, AP3B1, ARID1A, ARPC1B, ASXL1, ATG2B, ATM, ATR, ATRX, AXIN1, BCL11B, BCL2, BCL6, BCOR, BCORL1, BHLHE41, BIRC3, BLM, BPGM, BRAF, BRCA1, BRCA2, BRCC3, BRINP3, BRIP1, BTG1, BTK, BTLA, C3, C4BPA, C4BPB, CALN1, CALR, CARD11, CASP10, CBL, CBLB, CBLC, CCND1, CD200, CD247, CD27, CD36, CD3D, CD3E, CD40LG, CD46, CD58, CD59, CD79B, CDAN1, CDKN1B, CDKN2A, CDKN2B, CEBPA, CFB, CFH, CFHR1, CFHR3, CFHR4, CFHR5, CFI, CHD1, CHD4, CHD9, CHMP2B, CLPB, CNOT3, COX4I2, CREBBP, CRLF2, CSF1R, CSF2RA, CSF3R, CTC1, CTCF, CTSC, CUBN, CUX1, CXCR4, CYB5R3, CYBA, CYBB, CYCS, DCLRE1C, DDX41, DGKE, DGKH, DHFR, DIS3, DKC1, DNM2, DNMT1, DNMT3A, EBF1, ECT2L, EED, EGFR, EGLN1, EGLN2, EGLN3, EHMT1, ELANE, EP300, EPAS1, EPB41, EPB42, EPCAM, EPO, EPOR, ERCC4, ERG, ETNK1, ETV6, EZH2, F2R, FANCA, FANCB, FANCC, FANCD2, FANCE, FANCF, FANCG, FANCI, FANCL, FANCM, FAS, FASLG, FAT1, FBXW7, FCGR1A, FCGR3B, FERMT3, FLI1, FLNA, FLT3, FOXP3, G6PC3, G6PD, GATA1, GATA2, GATA3, GCLC, GFI1, GFI1B, GIF, GINS1, GLRX5, GNAS, GNB1, GP1BA, GP1BB, GP9, GPI, GPRC5A, GPX1, GSKIP, GSN, GSR, GSS, HAX1, HBA1, HBA2, HBB, HBD, HCLS1, HFE, HIF1A, HIF1AN, HIF3A, HK1, HNRNPK, HOOK1, HOXA10, HOXA11, HRAS, HSPA9, HUWE1, ID3, IDH1, IDH2, IFNG, IFNGR1, IFNGR2, IKZF1, IKZF2, IKZF3, IL12RB1, IL2RB, IL2RG, IL3RA, IL7R, IRF1, ITGA2, ITGA2B, ITGB2, ITGB3, ITK, ITPKB, JAGN1, JAK1, JAK2, JAK3, JAKMIP2, JMJD1C, KDM5C, KDM6A, KDM7A, KIF23, KIT, KLF1, KMT2A, KMT2C, KMT2D, KRAS, LAMB4, LAMTOR2, LAPTM5, LCK, LEF1, LIG4, LMNA, LMO1, LMO2, LPIN2, LRP1B, LRRC4, LUC7L2, LYL1, LYST, MAD2L2, MAGT1, MAP2K1, MAP2K2, MASTL, MBL2, MECOM, MED13, MEF2B, MEF2C, MEFV, MET, MLH1, MLLT10, MLLT3, MPL, MSH2, MSH4, MSH6, MTA1, MTAP, MTR, MTRR, MVK, MYB, MYC, MYD88, MYH9, MYSM1, NAF1, NBEAL2, NBN, NCF2, NCOR2, NF1, NHEJ1, NHP2, NLRP3, NOD2, NOP10, NOTCH1, NOTCH2, NPM1, NR3C1, NRAS, NT5C2, NT5C3A, NTRK3, NUP214, OS9, P2RY2, PALB2, PARN, PAX5, PBX1, PC, PCDHB1, PDGFRA, PDGFRB, PDHA1, PDHX, PFKL, PFKM, PGK1, PGM3, PHF6, PICALM, PIEZO1, PIGA, PIK3CD, PIK3R1, PKLR, PML, PMS2, PNP, POT1, PRDM1, PRF1, PRKACG, PRPF40B, PTCH2, PTEN, PTK2B, PTPN11, PTPN2, PTPRC, PTPRD, PUS1, RAB27A, RAC1, RAC2, RAD21, RAD50, RAD51, RAD51C, RAF1, RAG1, RAG2, RB1, RBBP6, RBM8A, RELN, RFWD3, RHAG, RHOA, RIT1, RMRP, RNF168, RPL10, RPL11, RPL15, RPL23, RPL26, RPL27, RPL31, RPL35A, RPL36, RPL5, RPS10, RPS14, RPS15, RPS17, RPS19, RPS24, RPS26, RPS27, RPS27A, RPS28, RPS29, RPS7, RTEL1, RUNX1, RUNX1T1, SAMD9L, SBDS, SBF2, SEC23B, SERPING1, SETBP1, SETD2, SF1, SF3A1, SF3B1, SH2B3, SH2D1A, SHOC2, SLC11A2, SLC19A2, SLC25A38, SLC2A1, SLC35C1, SLC37A4, SLC4A1, SLCO1B1, SLCO1B3, SLFN14, SLX4, SMARCD2, SMC1A, SMC3, SOS1, SPINK5, SPRED1, SPTA1, SPTB, SRC, SRCAP, SRP72, SRSF2, STAG1, STAG2, STAT3, STAT5B, STEAP3, STX11, STXBP2, SUZ12, SYNE1, TAL1, TAL2, TAZ, TBL1XR1, TBX1, TCF3, TCIRG1, TEC, TERC, TERF1, TERF2, TERF2IP, TERT, TET1, TET2, TET3, THBD, THPO, TINF2, TLX1, TLX3, TMPRSS6, TNFAIP3, TNFRSF13B, TNFRSF14, TNFRSF1A, TOX, TP53, TPI1, TPMT, TRAF3, TRNT1, TSLP, TSR2, TUBB1, TYK2, U2AF1, U2AF2, UBE2T, UGT1A1, UGT1A7, UNC13B, UNC13D, UNC5D, USB1, USH2A, USP9X, VHL, VPS13B, VPS45, VWF, WAS, WDR1, WIPF1, WRAP53, WT1, XBP1, XIAP, XK, XRCC2, YARS2, ZAP70, ZFHX4, ZNF197, ZRSR2, MRE11A, WHSC1, STON1, OBFC1*

1. 531 genes

*ABCB7, ABCG5, ABCG8, ABL1, ABL2, ACD, ACTB, ACTN1, ACVRL1, ADA, ADAMTS13, AIRE, AK1, AK2, AKT2, ALAS2, ALDOA, AMN, ANK1, ANKRD26, ANO6, AP3B1, ARID1A, ARPC1B, ASXL1, ATG2B, ATM, ATR, ATRX, AXIN1, BCL11B, BCL2, BCL6, BCOR, BCORL1, BIRC3, BLM, BLOC1S3, BLOC1S6, BPGM, BRAF, BRCA1, BRCA2, BRCC3, BRIP1, BTG1, BTK, BTLA, C15orf41, C1QA, C1QB, C1QC, C1R, C1S, C2, C3, C4A, C4B, C4BPA, C5, C6, C7, C8A, C8B, C9, CALR, CARD11, CASP10, CBL, CBLB, CCND1, CD200, CD247, CD27, CD36, CD3D, CD3E, CD40LG, CD46, CD58, CD59, CD79B, CDAN1, CDKN1B, CDKN2A, CDKN2B, CEBPA, CFB, CFD, CFH, CFHR1, CFHR2, CFHR3, CFHR4, CFHR5, CFI, CFP, CHD9, CLPB, CNOT3, COL3A1, COL4A1, COL4A2, COX4I2, CREBBP, CRLF2, CSF1R, CSF3R, CST3, CTC1, CTCF, CUBN, CUX1, CXCR4, CYB5R3, CYBA, CYBB, CYCS, DCLRE1C, DDX41, DGKE, DHFR, DIAPH1, DIS3, DKC1, DNAJC21, DNM2, DNMT1, DNMT3A, DPAGT1, DTNBP1, EBF1, ECT2L, EED, EFL1, EGFR, EGLN1, EGLN2, EIF2AK4, ELANE, ENG, EP300, EPAS1, EPB41, EPB42, EPCAM, EPO, EPOR, ERCC4, ERCC6L2, ERG, ETNK1, ETV6, EZH2, F10, F11, F12, F13A1, F13B, F2, F2R, F5, F7, F8, F9, FAM46C, FANCA, FANCB, FANCC, FANCD2, FANCE, FANCF, FANCG, FANCI, FANCL, FANCM, FAS, FASLG, FAT1, FBXW7, FCGR3B, FERMT3, FGA, FGB, FGG, FLI1, FLT3, FOXP3, FYB, G6PC3, G6PD, GATA1, GATA2, GATA3, GBA, GCLC, GFI1, GFI1B, GGCX, GIF, GINS1, GLA, GLRX5, GNAS, GNB1, GP1BA, GP1BB, GP6, GP9, GPI, GPRC5A, GPX1, GSR, GSS, HAX1, HBA1, HBA2, HBB, HBD, HFE, HIF1A, HK1, HOOK1, HOXA11, HPS1, HPS3, HPS4, HPS5, HPS6, HRAS, HRG, HSPA9, ID3, IDH1, IDH2, IFNG, IFNGR1, IKZF1, IKZF2, IKZF3, IL2RB, IL2RG, IL7R, IRF1, ITGA2, ITGA2B, ITGB2, ITGB3, ITK, JAGN1, JAK1, JAK2, JAK3, KCNK3, KDM5C, KDM6A, KIF23, KIT, KLF1, KLKB1, KMT2A, KMT2C, KMT2D, KNG1, KRAS, LAMB4, LAMTOR2, LCK, LIG4, LMAN1, LMNA, LMO1, LPIN2, LRP1B, LUC7L2, LYL1, LYST, MAD2L2, MAGT1, MAP2K1, MAP2K2, MASTL, MBL2, MCFD2, MECOM, MEF2B, MEFV, MET, MKL1, MLH1, MLPH, MMACHC, MMADHC, MPL, MRE11, MSH2, MSH6, MTAP, MTHFR, MTR, MTRR, MUT, MVK, MYB, MYC, MYD88, MYH9, MYO5A, MYSM1, NAF1, NBEAL2, NBN, NCF2, NCOR2, NF1, NFE2, NHEJ1, NHP2, NLRP3, NOP10, NOTCH1, NOTCH2, NPM1, NR3C1, NRAS, NSD2, NT5C2, NT5C3A, NTRK3, NUP214, P2RX1, P2RY12, PALB2, PARN, PAX5, PBX1, PDGFRA, PDGFRB, PDHA1, PDHX, PFKL, PFKM, PGK1, PGM3, PHF6, PICALM, PIEZO1, PIGA, PIK3CD, PIK3R1, PKLR, PLA2G4A, PLA2G7, PLAT, PLAU, PLG, PMS2, PNP, POT1, PPM1D, PRF1, PRKACG, PROC, PROCR, PROS1, PRPF40B, PTEN, PTK2B, PTPN11, PTPN2, PTPRC, PTPRD, PUS1, RAB27A, RAC1, RAC2, RAD21, RAD50, RAD51, RAD51C, RAF1, RAG1, RAG2, RASGRP2, RB1, RBBP6, RBM8A, RELN, RFWD3, RHAG, RHOA, RIT1, RPL10, RPL11, RPL15, RPL26, RPL27, RPL31, RPL35A, RPL36, RPL5, RPS10, RPS14, RPS15, RPS17, RPS19, RPS24, RPS26, RPS27, RPS27A, RPS28, RPS29, RPS7, RTEL1, RUNX1, RUNX1T1, SAMD9, SAMD9L, SBDS, SEC23B, SERPINC1, SERPIND1, SERPINE1, SERPINF2, SERPING1, SETBP1, SETD2, SF1, SF3A1, SF3B1, SH2B3, SH2D1A, SHOC2, SLC11A2, SLC19A2, SLC25A38, SLC2A1, SLC35C1, SLC37A4, SLC4A1, SLCO1B1, SLCO1B3, SLFN14, SLX4, SMAD4, SMAD9, SMARCD2, SMC1A, SMC3, SMPD1, SOS1, SPINK5, SPRED1, SPTA1, SPTB, SRC, SRP54, SRP72, SRSF2, STAG1, STAG2, STAT3, STAT5B, STEAP3, STIM1, STN1, STX11, STXBP2, SUZ12, SYNE1, TAL1, TAZ, TBL1XR1, TBX1, TBXA2R, TBXAS1, TCF3, TCIRG1, TEC, TERC, TERF2IP, TERT, TET1, TET2, TET3, TFPI, THBD, THPO, TINF2, TLX1, TLX3, TMPRSS6, TNFAIP3, TNFRSF13B, TNFRSF14, TNFRSF1A, TOX, TP53, TPI1, TRAF3, TREX1, TRNT1, TSLP, TSR2, TUBB1, TYK2, U2AF1, U2AF2, UBE2T, UGT1A1, UNC13D, USB1, VHL, VIPAS39, VKORC1, VPS13B, VPS33B, VPS45, VWF, WAS, WDR1, WIPF1, WRAP53, WT1, XBP1, XIAP, XK, XRCC2, YARS2, ZAP70, ZRSR2(531genes)*

**Supplementary Table S2.** Variants identified at diagnosis and relapse of acute myeloid leukemia patients.

| Patient # | NGS# | Diagnosis | Panel size | Gene | Transcript number | NT alteration | AA alteration | Depth | VAF (%) | Type | Tier |
| --- | --- | --- | --- | --- | --- | --- | --- | --- | --- | --- | --- |
| P1 | Diagnosis | Acute megakaryoblastic leukemia | 213 | *GATA1* | NM_002049.3 | c.137_164del | p.Ser46LeufsTer82 | 75 | 14.7 | Frameshift | Tier 1 |
|  |  |  |  | *EGLN1* | NM_022051.2 | c.2T>G | p.Met1? | 123 | 4.9 | Missense | Tier 3 |
|  |  |  |  | *EZH2* | NM_004456.4 | c.2050C>T | p.Arg684Cys | 329 | 5.8 | Missense | Tier 3 |
|  | Relapse |  | 213 | Not detected |  |  |  |  |  |  |  |
| P2 | Diagnosis | Acute monocytic leukemia | 213 | *KRAS* | NM_004985.3 | c.35G>A | p.Gly12Asp | 211 | 12.2 | Missense | Tier 2 |
|  | Relapse |  | 213 | *TP53* | NM_000546.5 | c.817C>T | p.Arg273Cys | 4330 | 9.1 | Missense | Tier 1 |
|  |  |  |  | *TP53* | NM_000546.5 | c.338T>C | p.Phe113Ser | 3586 | 26.5 | Missense | Tier 1 |
|  |  |  |  | *TP53* | NM_000546.5 | c.396G>C | p.Lys132Asn | 4633 | 15 | Missense | Tier 1 |
|  |  |  |  | *KRAS* | NM_004985.4 | c.35G>A | p.Gly12Asp | 2253 | 25.5 | Missense | Tier 2 |
|  |  |  |  | *BCORL1* | NM_021946.4 | c.3466C>T | p.Gln1156Ter | 1106 | 7.1 | Nonsense | Tier 2 |
|  |  |  |  | *ATM* | NM_000051.3 | c.9068G>A | p.Gly3023Asp | 1411 | 10.8 | Missense | Tier 3 |
|  |  |  |  | *CBL* | NM_005188.3 | c.1777C>T | p.Arg593Trp | 531 | 13.7 | Missense | Tier 3 |
|  |  |  |  | *ATR* | NM_001184.3 | c.1439G>T | p.Ser480Ile | 728 | 10.7 | Missense | Tier 3 |
| P3 | Diagnosis | Acute myeloid leukemia with maturation | 213 | *IDH2* | NM_002168.2 | c.419G>A | p.Arg140Gln | 3935 | 42.8 | Missense | Tier 1 |
|  |  |  |  | *NBN* | NM_002485.4 | c.982del | p.His328IlefsTer6 | 641 | 33.9 | Frameshift | Tier 2 |
|  | Relapse |  | 497 | *IDH2* | NM_002168.2 | c.419G>A | p.Arg140Gln | 1518 | 43 | Missense | Tier 1 |
|  |  |  |  | *NBN* | NM_002485.4 | c.982del | p.His328IlefsTer6 | 1430 | 38.7 | Frameshift | Tier 2 |
|  |  |  |  | *TCIRG1* | NM_006019.3 | c.931G>A | p.Val311Met | 997 | 5.2 | Missense | Tier 3 |
|  |  |  |  | *NUP214* | NM_005085.3 | c.4975_4989del | p.Gln1659_Asn1663del | 780 | 22.3 | Frameshift | Tier 3 |
| P4 | Diagnosis | Acute myeloid leukemia with mutated *NPM1* | 213 | *DNMT3A* | NM_022552.4 | c.2645G>A | p.Arg882His | 5617 | 47.4 | Missense | Tier 1 |
|  |  |  |  | *NPM1* | NM_002520.6 | c.860_863dup | p.Trp288CysfsTer12 | 2043 | 27.3 | Frameshift | Tier 1 |
|  |  |  |  | *FLT3* | NM_004119.2 | *FLT3*-ITD |  | 787 | 93.7 | Frameshift | Tier 1 |
|  | Relapse |  | 497 | *NPM1* | NM_002520.6 | c.860_863dup | p.Trp288CysfsTer12 | 880 | 23 | Frameshift | Tier 1 |
|  |  |  |  | *FLT3* | NM_004119.2 | FLT3-ITD |  | 450 | 80.7 | Frameshift | Tier 1 |
|  |  |  |  | *DNMT3A* | NM_022552.4 | c.2645G>A | p.Arg882His | 1804 | 47.6 | Missense | Tier 1 |
|  |  |  |  | *WT1* | NM_024426.4 | c.1133_1142dup | p.Ala382CysfsTer6 | 462 | 33.9 | Frameshift | Tier 1 |
|  |  |  |  | *WT1* | NM_024426.4 | c.1138delCinsGG | p.Arg380GlyfsTer5 | 351 | 15.6 | Frameshift | Tier 1 |
| P5 | Diagnosis | Acute myeloid leukemia with mutated NPM1 | 213 | *FLT3* | NM_004119.2 | *FLT3*-ITD |  | 2711 | 53.8 | Frameshift | Tier 1 |
|  |  |  |  | *NPM1* | NM_002520.6 | c.860_863dup | p.Trp288CysfsTer12 | 2230 | 40.2 | Frameshift | Tier 1 |
|  |  |  |  | *FANCE* | NM_021922.2 | c.1316G>A | p.Gly439Glu | 125 | 48 | Missense | Tier 3 |
|  |  |  |  | *KIT* | NM_000222.2 | c.56G>A | p.Arg19His | 118 | 47.5 | Missense | Tier 3 |
|  | Relapse |  | 213 | *FLT3* | NM_004119.2 | *FLT3*-ITD |  | 1299 | 68.4 | Frameshift | Tier 1 |
|  |  |  |  | *NPM1* | NM_002520.6 | c.860_863dup | p.Trp288CysfsTer12 | 1059 | 37 | Frameshift | Tier 1 |
| P6 | Diagnosis | Acute myeloid leukemia with t(16;16)(p13.1;q22) | 497 | Not detected |  |  |  |  |  |  |  |
|  | Relapse |  | 497 | *KIT* | NM_000222.2 | c.2447A>T | p.Asp816Val | 2865 | 44.8 | Missense | Tier 2 |
| P7 | Diagnosis | Acute monocytic leukemia | 497 | *FLT3* | NM_004119.2 | *FLT3*-ITD |  | 1087 | 18.3 | Frameshift | Tier 1 |
|  |  |  |  | *RUNX1* | NM_001754.4 | c.339_340insTCTC | p.Ile114SerfsTer25 | 759 | 17.5 | Frameshift | Tier 1 |
|  |  |  |  | *RUNX1* | NM_001754.4 | c.422C>T | p.Ser141Leu | 1139 | 25.6 | Missense | Tier 3 |
|  | Relapse |  | 497 | *FLT3* | NM_004119.2 | *FLT3*-ITD |  | 744 | 20.6 | Frameshift | Tier 1 |
|  |  |  |  | *RUNX1* | NM_001754.4 | c.339_340insTCTC | p.Ile114SerfsTer25 | 956 | 21.7 | Frameshift | Tier 1 |
|  |  |  |  | *BCORL1* | NM_021946.4 | c.3368_3392del | p.Lys1123SerfsTer91 | 1244 | 8.6 | Frameshift | Tier 2 |
|  |  |  |  | *RUNX1* | NM_001754.4 | c.422C>T | p.Ser141Leu | 466 | 28.2 | Missense | Tier 3 |
|  |  |  |  | *NCOR2* | NM_006312.5 | c.3679G>A | p.Gly1227Ser | 1168 | 16.7 | Missense | Tier 3 |
| P8 | Diagnosis | Acute myeloid leukemia with t(8;21)(q22;q22.1) | 497 | *KIT* | NM_000222.2 | c.2466T>G | p.Asn822Lys | 1413 | 32.4 | Missense | Tier 1 |
|  |  |  |  | *WT1* | NM_024426.4 | c.1109_1110insC | p.Val371CysfsTer14 | 1045 | 8.4 | Frameshift | Tier 2 |
|  | Relapse |  | 497 | *KIT* | NM_000222.2 | c.2466T>G | p.Asn822Lys | 2251 | 29.6 | Missense | Tier 1 |
|  |  |  |  | *WT1* | NM_024426.4 | c.1372delinsGG | p.Arg458GlyfsTer19 | 1834 | 26.7 | Frameshift | Tier 2 |
|  |  |  |  | *GATA2* | NM_001145661.1 | c.1087_1088insCGCGAA | p.Arg362_Asn363insThrArg | 1795 | 20.3 | Missense | Tier 3 |
|  |  |  |  | *GNAS* | NM_001077490.1 | c.322C>G | p.Pro108Ala | 2365 | 19.5 | Missense | Tier 3 |
|  |  |  |  | *FANCM* | NM_020937.2 | c.424C>T | p.Pro142Ser | 2012 | 26.5 | Missense | Tier 3 |
|  |  |  |  | *NOTCH1* | NM_017617.3 | c.3008A>G | p.Asn1003Ser | 2162 | 28.7 | Missense | Tier 3 |
|  |  |  |  | *IRF1* | NM_002198.2 | c.845G>T | p.Ser282Ile | 974 | 5 | Missense | Tier 3 |
|  |  |  |  | *NBEAL2* | NM_015175.2 | c.8219A>G | p.Gln2740Arg | 2269 | 32.2 | Missense | Tier 3 |
| P9 | Diagnosis | Acute monocytic leukemia | 497 | *NPM1* | NM_002520.6 | c.860_863dup | p.Trp288CysfsTer12 | 812 | 30.9 | Frameshift | Tier 1 |
|  |  |  |  | *IDH2* | NM_002168.3 | c.419G>A | p.Arg140Gln | 1010 | 45.1 | Missense | Tier 1 |
|  |  |  |  | *DNMT3A* | NM_022552.4 | c.2129G>A | p.Cys710Tyr | 890 | 43.4 | Missense | Tier 2 |
|  |  |  |  | *PTPN11* | NM_002834.3 | c.227A>C | p.Glu76Ala | 720 | 6.7 | Missense | Tier 2 |
|  |  |  |  | *PTPN11* | NM_002834.3 | c.181G>C | p.Asp61His | 577 | 7.6 | Missense | Tier 2 |
|  |  |  |  | *NRAS* | NM_002524.4 | c.38G>A | p.Gly13Asp | 974 | 8.1 | Missense | Tier 2 |
|  | Relapse |  | 497 | *FLT3* | NM_004119.2 | *FLT3*-ITD |  | 1088 | 17.2 | Frameshift | Tier 1 |
|  |  |  |  | *NPM1* | NM_002520.6 | c.860_863dup | p.Trp288CysfsTer12 | 1898 | 13.6 | Frameshift | Tier 1 |
|  |  |  |  | *IDH2* | NM_002168.2 | c.419G>A | p.Arg140Gln | 2985 | 27.3 | Missense | Tier 1 |
|  |  |  |  | *DNMT3A* | NM_022552.4 | c.2129G>A | p.Cys710Tyr | 2576 | 29.4 | Missense | Tier 2 |
| P10 | Diagnosis | Acute myeloid leukemia with t(8;21)(q22;q22.1) | 497 | *RUNX1* |  | partial deletion |  |  |  | CNV | Tier 2 |
|  |  |  |  | *KRAS* | NM_004985.4 | c.38G>A | p.Gly13Asp | 1583 | 24.8 | Missense | Tier 2 |
|  |  |  |  | *STAG2* | NM_001042749.2 | c.2469dup | p.Glu824Ter | 408 | 83.6 | Nonsense | Tier 2 |
|  |  |  |  | *CSF1R* | NM_005211.3 | c.1967G>A | p.Gly656Glu | 638 | 39.5 | Missense | Tier 3 |
|  | Relapse |  | 497 | *STAG2* | NM_001042749.1 | c.2469dup | p.Glu824Ter | 519 | 23.5 | Nonsense | Tier 2 |
|  |  |  |  | *CSF1R* | NM_005211.3 | c.1967G>A | p.Gly656Glu | 778 | 11.7 | Missense | Tier 3 |
|  |  |  |  | *CLPB* | NM_030813.4 | c.1592G>A | p.Ser531Asn | 871 | 17.1 | Missense | Tier 3 |
| P11 | Diagnosis | Acute myeloid leukemia with biallelic mutations of *CEBPA* | 497 | *CEBPA* | NM_004364.3 | c.68del | p.Pro23ArgfsTer137 | 957 | 34.8 | Frameshift | Tier 1 |
|  |  |  |  | *CEBPA* | NM_004364.4 | c.934_936dup | p.Gln312dup | 1226 | 33 | Frameshift | Tier 1 |
|  |  |  |  | *EZH2* | NM_004456.4 | Whole gene deletion |  |  |  | CNV | Tier 3 |
|  |  |  |  | *EZH2* | NM_004456.4 | c.2191T>C | p.Tyr731His | 517 | 34.6 | Missense | Tier 3 |
|  | Relapse |  | 497 | *CEBPA* | NM_004364.3 | c.934_936dup | p.Gln312dup | 2310 | 19 | Missense | Tier 1 |
|  |  |  |  | *CEBPA* | NM_004364.3 | c.68del | p.Pro23ArgfsTer137 | 1891 | 16.7 | Frameshift | Tier 1 |
|  |  |  |  | *EZH2* | NM_004456.4 | c.2191T>C | p.Tyr731His | 921 | 18.9 | Missense | Tier 3 |
| P12 | Diagnosis | Acute myeloid leukemia with maturation | 497 | *NRAS* | NM_002524.4 | c.35G>A | p.Gly12Asp | 1589 | 15.3 | Missense | Tier 2 |
|  |  |  |  | *HNRNPK* | NM_002140.3 | c.589G>A | p.Gly197Arg | 987 | 24.8 | Missense | Tier 3 |
|  |  |  |  | *RUNX1* | NM_001754.4 | c.1274C>G | p.Pro425Arg | 432 | 25.5 | Missense | Tier 3 |
|  | Relapse |  | 497 | *FANCF* | NM_022725.3 | c.1009G>T | p.Gly337Ter | 655 | 5.6 | Nonsense | Tier 3 |
|  |  |  |  | *SETBP1* | NM_015559.2 | c.4563C>A | p.Pro1521= | 910 | 22.7 | Splicing | Tier 3 |
|  |  |  |  | *HNRNPK* | NM_002140.4 | c.589G>A | p.Gly197Arg | 763 | 30 | Missense | Tier 3 |
|  |  |  |  | *MSH6* | NM_000179.2 | c.3245C>T | p.Pro1082Leu | 922 | 30.3 | Missense | Tier 3 |
| P13 | Diagnosis | Acute myeloid leukemia with maturation | 497 | *RIT1* | NM_006912.5 | c.244T>A | p.Phe82Ile | 621 | 34.5 | Missense | Tier 2 |
|  |  |  |  | *NPM1* | NM_002520.6 | c.408_409insGTGGAGGATGTGAAACTCTTA | p.Leu136_Ser137insValGluAspValLysLeuLeu | 495 | 13.7 | Indel | Tier 3 |
|  |  |  |  | *NOTCH2* | NM_024408.3 | c.7G>A | p.Ala3Thr | 984 | 31.5 | Missense | Tier 3 |
|  | Relapse |  | 531 | *RIT1* | NM_006912.6 | c.244T>A | p.Phe82Ile | 971 | 11.5 | Missense | Tier 2 |
|  |  |  |  | *DNMT3A* | NM_022552.5 | c.2146G>T | p.Val716Phe | 1487 | 24.8 | Missense | Tier 3 |
|  |  |  |  | *NPM1* | NM_002520.7 | c.408_409insGTGGAGGATGTGAAACTCTTA | p.Leu136_Ser137insValGluAspValLysLeuLeu | 839 | 9.8 | Indel | Tier 3 |
|  |  |  |  | *NOTCH2* | NM_024408.4 | c.7G>A | p.Ala3Thr | 2074 | 25.4 | Missense | Tier 3 |
| P14 | Diagnosis | Acute myeloid leukemia with mutated NPM1 | 497 | *FLT3* | NM_004119.2 | *FLT3*-ITD |  | 1262 | 18.6 | Frameshift | Tier 1 |
|  |  |  |  | *NPM1* | NM_002520.6 | c.860_863dup | p.Trp288CysfsTer12 | 934 | 28.8 | Frameshift | Tier 2 |
|  |  |  |  | *TET2* | NM_001127208.2 | c.1526C>G | p.Ser509Ter | 1027 | 49.4 | Nonsense | Tier 2 |
|  | Relapse |  | 497 | *FLT3* | NM_004119.2 | *FLT3*-ITD |  | 1327 | 23 | Frameshift | Tier 1 |
|  |  |  |  | *NPM1* | NM_002520.6 | c.860_863dup | p.Trp288CysfsTer12 | 1086 | 20.1 | Frameshift | Tier 2 |
|  |  |  |  | *TET2* | NM_001127208.2 | c.1526C>G | p.Ser509Ter | 1263 | 41.6 | Nonsense | Tier 2 |
|  |  |  |  | *RUNX1* | NM_001754.4 | c.879_880del | p.Pro294PhefsTer305 | 1437 | 9.9 | Frameshift | Tier 2 |
| P15 | Diagnosis | Acute myelomonocytic leukemia | 497 | *ASXL1* | NM_015338.5 | c.2644C>T | p.Gln882Ter | 702 | 35.5 | Nonsense | Tier 2 |
|  |  |  |  | *NF1* | NM_001042492.2 | c.5907_5908del | p.Arg1970SerfsTer6 | 917 | 33.4 | Frameshift | Tier 2 |
|  |  |  |  | *VWF* | NM_000552.4 | c.3797C>A | p.Pro1266Gln | 631 | 8.7 | Missense | Tier 3 |
|  |  |  |  | *IRF1* | NM_002198.2 | c.697_717+5del |  | 481 | 12.1 | Frameshift | Tier 3 |
|  | Relapse |  | 531 | *ASXL1* | NM_015338.5 | c.2644C>T | p.Gln882Ter | 1865 | 23.9 | Nonsense | Tier 2 |
|  |  |  |  | *NF1* | NM_001042492.2 | c.5907_5908del | p.Arg1970SerfsTer6 |  | 2.6 | Frameshift | Tier 2 |
|  |  |  |  | *BRCA2* | NM_000059.3 | c.97G>A | p.Glu33Lys | 1155 | 22.3 | Missense | Tier 3 |
|  |  |  |  | *ID3* | NM_002167.4 | c.275del | p.Pro92LeufsTer34 | 1686 | 22.4 | Frameshift | Tier 3 |
|  |  |  |  | *IRF1* | NM_002198.2 | c.697_717+5del |  | 1544 | 18.5 | Frameshift | Tier 3 |
|  |  |  |  | *CBL* | NM_005188.3 | c.1192C>T | p.His398Tyr | 1312 | 39.9 | Missense | Tier 3 |
| P16 | Diagnosis | Acute myeloid leukemia with t(8;21)(q22;q22.1) | 497 | *KIT* | NM_000222.2 | c.2447A>T | p.Asp816Val | 3795 | 24.7 | Missense | Tier 1 |
|  |  |  |  | *U2AF1* | NM_001025203.1 | c.101C>A | p.Ser34Tyr | 2440 | 21.1 | Missense | Tier 2 |
|  | Relapse |  | 497 | *KIT* | NM_000222.2 | c.2447A>T | p.Asp816Val | 4415 | 58.1 | Missense | Tier 1 |
|  |  |  |  | *U2AF1* | NM_001025203.1 | c.101C>A | p.Ser34Tyr | 2098 | 35.9 | Missense | Tier 2 |
|  |  |  |  | *ADAMTS13* | NM_139025.4 | c.3541G>A | p.Gly1181Arg | 1310 | 9.5 | Missense | Tier 3 |
|  |  |  |  | *BLM* | NM_000057.3 | c.2656C>A | p.His886Asn | 2574 | 6.1 | Missense | Tier 3 |
| P17 | Diagnosis | Acute myelomonocytic leukemia | 497 | *TET2* |  | Whole gene deletion |  |  |  | CNV | Tier 2 |
|  |  |  |  | *TET2* | NM_001127208.2 | c.1835del | p.Pro612LeufsTer27 | 1346 | 87 | Frameshift | Tier 2 |
|  |  |  |  | *ZRSR2* | NM_005089.3 | c.868C>T | p.Arg290Ter | 562 | 85.3 | Nonsense | Tier 2 |
|  |  |  |  | *CBL* | NM_005188.3 | c.1111T>C | p.Tyr371His | 1641 | 65 | Missense | Tier 3 |
|  | Relapse |  | 531 | *TET2* |  | Whole gene deletion |  |  |  | CNV | Tier 2 |
|  |  |  |  | *TET2* | NM_001127208.2 | c.1835del | p.Pro612LeufsTer27 | 1081 | 82.8 | Frameshift | Tier 2 |
|  |  |  |  | *ZRSR2* | NM_005089.3 | c.868C>T | p.Arg290Ter | 847 | 89 | Nonsense | Tier 2 |
|  |  |  |  | *CBL* | NM_005188.3 | c.1111T>C | p.Tyr371His | 1377 | 89.8 | Missense | Tier 3 |
|  |  |  |  | *ZRSR2* | NM_005089.3 | c.1302_1303insATGGACCGCAGC | p.Ser434_Arg435insMetAspArgSer | 613 | 82.4 | Indel | Tier 3 |
| P18 | Diagnosis | Acute monocytic leukemia | 497 | *FLT3* | NM_004119.2 | *FLT3*-ITD |  | 2031 | 14.7 | Frameshift | Tier 1 |
|  |  |  |  | *NPM1* | NM_002520.6 | c.860_863dup | p.Trp288CysfsTer12 | 1950 | 24.8 | Frameshift | Tier 2 |
|  |  |  |  | *DNMT3A* | NM_022552.4 | c.2644C>T | p.Arg882Cys | 2759 | 46.6 | Frameshift | Tier 2 |
|  | Relapse |  | 531 | *FLT3* | NM_004119.2 | *FLT3*-ITD |  | 2268 | 53.6 | Frameshift | Tier 1 |
|  |  |  |  | *NPM1* | NM_002520.6 | c.860_863dup | p.Trp288CysfsTer12 | 1517 | 29.8 | Frameshift | Tier 2 |
|  |  |  |  | *DNMT3A* | NM_022552.4 | c.2644C>T | p.Arg882Cys | 2301 | 35.7 | Missense | Tier 2 |
|  |  |  |  | *FANCB* | NM_001018113.1 | c.1327-3dup |  | 388 | 5.2 | Splicing | Tier 3 |
|  |  |  |  | *VKORC1* | NM_024006.5 | c.95C>T | p.Ala32Val | 2851 | 39.4 | Missense | Tier 3 |
|  |  |  |  | *LPIN2* | NM_014646.2 | c.1536A>G | p.Ile512Met | 1356 | 11.1 | Missense | Tier 3 |
| P19 | Diagnosis | Acute myeloid leukemia with t(8;21)(q22;q22.1) | 497 | *ASXL1* | NM_015338.5 | c.1900_1922del | p.Glu635ArgfsTer15 | 1827 | 20.1 | Frameshift | Tier 1 |
|  |  |  |  | *PHF6* | NM_001015877.1 | c.73_74del | p.Asp25GlnfsTer10 | 2013 | 7.3 | Frameshift | Tier 2 |
|  |  |  |  | *JAK1* | NM_002227.2 | c.1954T>C | p.Tyr652His | 1639 | 7.1 | Missense | Tier 3 |
|  | Relapse |  | 531 | Not detected |  |  |  |  |  |  |  |
| P20 | Diagnosis | Acute myeloid leukemia with mutated *NPM1* | 497 | *FLT3* | NM_004119.2 | *FLT3*-ITD |  | 1539 | 11.8 | Frameshift | Tier 1 |
|  |  |  |  | *DNMT3A* | NM_022552.4 | c.2645G>A | p.Arg882His | 2461 | 33 | Missense | Tier 2 |
|  | Relapse |  | 531 | *FLT3* | NM_004119.2 | *FLT3*-ITD |  | 192 | 88 | Frameshift | Tier 1 |
|  |  |  |  | *DNMT3A* | NM_022552.4 | c.2645G>A | p.Arg882His | 279 | 48.4 | Missense | Tier 2 |
|  |  |  |  | *NPM1* | NM_002520.6 | c.860_863dup | p.Trp288CysfsTer12 | 271 | 42.4 | Frameshift | Tier 2 |
| P21 | Diagnosis | Acute myeloid leukemia with maturation | 497 | Not detected |  |  |  |  |  |  |  |
|  | Relapse |  | 531 | *RUNX1* | NM_001754.4 | Whole gene amplification |  |  |  | CNV | Tier 2 |
| P22 | Diagnosis | Acute myeloid leukemia with biallelic mutations of *CEBPA* | 497 | *CEBPA* | NM_004364.3 | c.67_68dup | p.His24ArgfsTer137 | 308 | 26.6 | Frameshift | Tier 1 |
|  |  |  |  | *CEBPA* | NM_004364.3 | c.934_936dup | p.Gln312dup | 758 | 40.9 | Frameshift | Tier 1 |
|  |  |  |  | *RAD21* | NM_006265.2 | c.355C>T | p.Gln119Ter | 354 | 32.2 | Nonsense | Tier 2 |
|  |  |  |  | *WT1* | NM_024426.4 | c.1400_1402dup | p.Lys467dup | 436 | 9.2 | Frameshift | Tier 3 |
|  |  |  |  | *WT1* | NM_024426.4 | c.1397_1399dup | p.Leu466_Lys467insMet | 433 | 8.8 | Frameshift | Tier 3 |
|  |  |  |  | *SETD2* | NM_014159.6 | c.4687G>T | p.Gly1563Cys | 418 | 5.5 | Missense | Tier 3 |
|  | Relapse |  | 531 | *CEBPA* | NM_004364.3 | c.67_68dup | p.His24ArgfsTer137 | 3073 | 42.3 | Frameshift | Tier 1 |
|  |  |  |  | *CEBPA* | NM_004364.3 | c.934_936dup | p.Gln312dup | 3076 | 47.5 | Frameshift | Tier 1 |
|  |  |  |  | *WT1* | NM_024426.4 | c.574_581delinsT | p.Gln192Ter | 1669 | 51.4 | Frameshift | Tier 2 |
|  |  |  |  | *CDKN2A* | NM_000077.4 | c.186del | p.Leu63CysfsTer83 | 2085 | 46 | Frameshift | Tier 2 |
|  |  |  |  | *WT1* | NM_024426.4 | c.1397_1399dup | p.Leu466_Lys467insMet | 2018 | 46.3 | Indel | Tier 3 |
|  |  |  |  | *KMT2D* | NM_003482.3 | c.9979C>G | p.Gln3327Glu | 2237 | 42.6 | Missense | Tier 3 |
|  |  |  |  | *SERPINF2* | NM_000934.3 | c.1169G>C | p.Gly390Ala | 2719 | 41.4 | Missense | Tier 3 |
|  |  |  |  | *BLM* | NM_000057.2 | c.492G>T | p.Glu164Asp | 1727 | 17.5 | Missense | Tier 3 |
|  |  |  |  | *JAK3* | NM_000215.3 | c.2570T>A | p.Leu857Gln | 1144 | 49.1 | Missense | Tier 3 |
|  |  |  |  | *LRP1B* | NM_018557.2 | c.13256C>G | p.Thr4419Ser | 1764 | 43.7 | Missense | Tier 3 |
| P23 | Diagnosis | Acute monocytic leukemia | 531 | *FLT3* | NM_004119.2 | FLT3-ITD |  | 2080 | 8.8 | Frameshift | Tier 1 |
|  |  |  |  | *KMT2A* | NM_001197104 | KMT2A-PTD |  |  |  | CNV | Tier 1 |
|  |  |  |  | *WT1* | NM_024426.4 | c.1128_1137dup | p.Arg380AspfsTer8 | 1379 | 28.3 | Frameshift | Tier 2 |
|  |  |  |  | *DNMT3A* | NM_022552.4 | c.1880del | p.Pro627GlnfsTer24 | 1812 | 43.7 | Frameshift | Tier 2 |
|  |  |  |  | *KMT2C* | NM_170606.2 | c.850-7C>T |  | 3663 | 13.1 | Splicing | Tier 3 |
|  | Relapse |  | 531 | *FLT3* | NM_004119.2 | *FLT3*-ITD |  | 2587 | 10.7 | Frameshift | Tier 1 |
|  |  |  |  | *KMT2A* | NM_001197104 | *KMT2A*-PTD |  |  |  | CNV | Tier 1 |
|  |  |  |  | *WT1* | NM_024426.4 | c.1128_1137dup | p.Arg380AspfsTer8 | 1694 | 13.6 | Frameshift | Tier 2 |
|  |  |  |  | *DNMT3A* | NM_022552.4 | c.1880del | p.Pro627GlnfsTer24 | 1765 | 17.1 | Frameshift | Tier 2 |
|  |  |  |  | *MTR* | NM_000254.2 | c.1542A>T | p.Arg514Ser | 1551 | 30.3 | Missense | Tier 3 |
|  |  |  |  | *MRE11* | NM_005591.3 | c.*3T>C |  | 1117 | 30.2 | Splicing | Tier 3 |
|  |  |  |  | *GCLC* | NM_001498.3 | c.493G>T | p.Val165Leu | 1767 | 33.7 | Missense | Tier 3 |
|  |  |  |  | *ALDOA* | NM_000034.3 | c.202G>A | p.Asp68Asn | 1588 | 31.3 | Missense | Tier 3 |
| P24 | Diagnosis | Acute myeloid leukemia with maturation | 531 | *DNMT3A* | NM_022552.4 | c.958C>T | p.Arg320Ter | 1279 | 25.2 | Nonsense | Tier 2 |
|  |  |  |  | *DNMT3A* | NM_022552.4 | c.1743G>A | p.Trp581Ter | 1125 | 29 | Nonsense | Tier 2 |
|  |  |  |  | *IDH2* | NM_002168.2 | c.419G>A | p.Arg140Gln | 1602 | 25 | Missense | Tier 2 |
|  |  |  |  | *PHF6* | NM_001015877.1 | c.385C>T | p.Arg129Ter | 846 | 20.9 | Nonsense | Tier 2 |
|  |  |  |  | *PGK1* | NM_000291.3 | c.1161T>A | p.Asp387Glu | 1052 | 25.9 | Missense | Tier 3 |
|  |  |  |  | *C6* | NM_000065.3 | c.2068G>A | p.Gly690Arg | 967 | 21.7 | Missense | Tier 3 |
|  | Relapse |  | 531 | *PHF6* | NM_001015877.2 | c.385C>T | p.Arg129Ter | 1095 | 30.2 | Nonsense | Tier 2 |
|  |  |  |  | *IDH2* | NM_002168.4 | c.419G>A | p.Arg140Gln | 2688 | 24 | Missense | Tier 2 |
|  |  |  |  | *DNMT3A* | NM_022552.5 | c.958C>T | p.Arg320Ter | 2791 | 18.8 | Nonsense | Tier 2 |
|  |  |  |  | *DNMT3A* | NM_022552.5 | c.1743G>A | p.Trp581Ter | 2370 | 36.9 | Nonsense | Tier 2 |
|  |  |  |  | *MSH6* | NM_000179.3 | c.3379G>A | p.Ala1127Thr | 2239 | 3.8 | Missense | Tier 3 |
|  |  |  |  | *VPS33B* | NM_018668.5 | c.403+2T>A |  | 1560 | 23 | Splicing | Tier 3 |
|  |  |  |  | *TNFRSF13B* | NM_012452.3 | c.740C>T | p.Thr247Met | 2532 | 24.2 | Missense | Tier 3 |
